# Computational detection of significant variation in binding affinity across two sets of sequences with application to the analysis of replication origins in yeast

**DOI:** 10.1186/1471-2105-9-372

**Published:** 2008-09-12

**Authors:** Uri Keich, Hong Gao, Jeffrey S Garretson, Anand Bhaskar, Ivan Liachko, Justin Donato, Bik K Tye

**Affiliations:** 1 Department of Computer Science, Cornell University, Ithaca, NY, 14853, USA; 2 Department of Biological Statistics & Computational Biology, Cornell University, Ithaca, NY, 14853, USA; 3 Department of Molecular Biology & Genetics, Cornell University, Ithaca, NY, 14853, USA; 4 Department of Bacteriology, University of Wisconsin, Madison, WI, 53706, USA

## Abstract

**Background:**

In analyzing the stability of DNA replication origins in *Saccharomyces cerevisiae *we faced the question whether one set of sequences is significantly enriched in the number and/or the quality of the matches of a particular position weight matrix relative to another set.

**Results:**

We present SADMAMA, a computational solution to a address this problem. SADMAMA implements two types of statistical tests to answer this question: one type is based on simplified models, while the other relies on bootstrapping, and as such might be preferable to users who are averse to such models. The bootstrap approach incorporates a novel "site-protected" resampling procedure which solves a problem we identify with naive resampling.

**Conclusion:**

SADMAMA's utility is demonstrated here by offering a plausible explanation to the differential ARS activity observed in our previous *mcm1-1 *mutant experiments [1], by suggesting the relevance of multiple weak ACS matches to efficient replication origin function in *Saccharomyces cerevisiae*, and by suggesting an explanation to the observed negative effect *FKH2 *has on chromatin silencing [2]. SADMAMA is available for download from .

## Background

In analyzing the stability of DNA replication origins in *S. cerevisiae *(see Stable vs. unstable ARSs in *mcm1-1 *mutant below) we faced the question of whether one set of sequences has more and/or better binding sites of a particular transcription factor than the other. One way to address this question is through wet lab experiments such as chromatin immunoprecipitation. Here we offer a computational alternative, which can be effective provided the PWM (position weight matrix, e.g. [3]) representation of the transcription factor is known. An obvious advantage of our computational approach is that it is much cheaper to execute and it provides a built-in statistical significance analysis.

There are many computational tools that scan for "good" matches of a given PWM (e.g., [4-7]). Similarly there are tools that look at the significance of PWM matches in a set of sequences (e.g., [8]). None of these however directly apply to our problem, where the null assumption is that there is "essentially" no difference in the binding sites between the two input sets, even though both might be enriched, deficient, or neutral in sites when compared to "background" sequences. Elkon et al. look for enrichment in the number of sites in a subset of a genomic scale set of promoters [9]. In particular their approach is not applicable when the sets of sequences are either disjoint or small. There has also been work on discriminative de-novo motif finding (e.g., [10]) where the goal is to *find *a PWM that discriminates between two sets of sequences. This is quite different from our stated goal where the PWM is given and the focus is on assigning significance to the difference in the number and/or quality of sites. Robin et al. study a very similar problem to ours, only in the context of a pattern representation of the motif [11]. In Conclusions section we emphasize some of the differences between this paper and their theoretical work. Here we present SADMAMA (Significance Assessment of the Difference in MAtrix MAtches) – the tool we have developed to address the aforementioned problem. SADMAMA implements two different strategies for testing the difference in site frequency as well as site quality between the two input sets. The quicker approach relies on a couple of simplified statistical models from which we derive and carefully implement the appropriate tests. As an alternative for accepting our simplified models, we offer bootstrapping which, by its nature, requires fewer assumptions, but consumes more time. The development of our bootstrap procedure required some innovation since, as we show below, a naive resampling approach can create false positives. That is, it can indicate a significant difference between two input sets that are essentially equivalent as far as the PWM sites are concerned.

Our motivation for developing SADMAMA came from our study of replication origins in *S. cerevisiae *(reviewed in [12]). DNA replication is a fundamental process essential for cell proliferation. While the proteins that are involved in initiating DNA replication are essentially conserved from yeast to humans, the implicated sequence motifs that these conserved factors interact with are poorly understood outside of *S. cerevisiae *([13,12]). Moreover, even for *S. cerevisiae *the replication initiation process is not completely understood. For example, it is known that the roughly 400 replication origins in *S. cerevisiae *([14-16]), called ARSs (Autonomously Replicating Sequences), differ in several important aspects from one another. These include timing and efficiency of origin firing, as well as sensitivity to mutations in proteins involved in replication initiation. However, much of this variability is yet to be explained and this is an active area of research. Our study in [1] was designed to identify ARSs that are preferentially used in yeast strains defective for replication initiation. SADMAMA was specifically designed to suggest sequence motifs that might explain the preferential usage we observed in [1]. Such information could help us gain insight into the determinants that regulate replication origin usage. Given our motivation for SADMAMA's development, it is fitting that we demonstrate its utility in that context:

• We show that SADMAMA provides a possible explanation for the difference in replication efficiency among two sets of ARSs we identified in [1].

• Essential to replication initiation is the binding of the ORC (Origin Recognition Complex) to the ACS (ARS Consensus Sequence) [17]. Using a screen for fragments of *S. kluyveri *DNA that have ARS function in *S. cerevisiae*, we provide evidence that support a recent conjecture that ORC binding in some *S. cerevisiae *ARSs requires multiple, seemingly redundant ACS matches [18].

• Finally, we demonstrate how SADMAMA can be used for exploratory data analysis.

## Results and Discussion

### Statistical Models and Tests

#### Scoring words and identifying sites

Since our goal is to assess the difference between the PWM matches in the two input sets we first need to define what we consider as a match. In order to do so we first need to specify how we score each putative site, or word of length *l*, where *l *is the length, or width, of the PWM. We use the log-likelihood ratio score $s(w)=\log\frac{p_{M}(w)}{p_{0}(w)}$ where $p_{M}(w)=\prod_{i=1}^{l}M_{iw_{i}}$ and *M*_*ij *_is the frequency of letter *j *in position *i *of the motif, and *p*_0_(*w*) is the null likelihood of *w*. Note that *M *here represents the PWM as a PFM (Position Frequency Matrix). In this paper we will generally not make the distinction between the two. Also note that given that our null model is a Markov chain the annotation *p*_0_(*w*) is somewhat misleading as this probability typically depends on the few characters preceding *w *in the sequence.

A word *w *is considered a match if its score exceeds a user specified threshold. For example, only words whose scores lie in the top 0.1% of the null scores are considered matches (in practice this threshold is determined using an appropriate null training model). While this defines whether a single word *w *is considered a match or not, we would often hesitate to consider two matches that almost completely overlap as two distinct matches. Here again we rely on the user to specify the amount of overlap that is tolerated between distinct sites, and we apply a greedy strategy to choose sites that conform to the specified overlap.

#### Measuring the difference in the number of sites: The binomial model

To assign significance to the site-frequency difference between the sets, we assume that matches (sites) occur in each of the sets according to a binomial model *B*(*n*_*i*_, *p*_*i*_) *i *= 1, 2, where *n*_*i *_is the number of possible sites in the corresponding set (roughly the set length), and *p*_*i *_is the site frequency. Clearly this simplistic model glosses over the problem of dependence between overlapping sites. However, overlap is not a real issue with most PWMs given a reasonably high threshold. Such thresholds are typically chosen anyhow, as binding sites are meant to be rather rare.

The null hypothesis *H*_0 _is that, *p*_1 _= *p*_2_. Note that this is different from the "null background", which specifies how the background is generated. In particular, *H*_0 _does not assume that all matches are merely random background matches, rather that they are some mixture of random background matches and "real sites". The alternative hypothesis can be a two sided *p*_1 _≠ *p*_2 _or a one sided *p*_1 _> *p*_2 _or *p*_2 _> *p*_1_. Assuming our binomial model, we can readily test for violation of the null assumption based on the fact that conditioned on the joint number of matches, the number of matches in the first set has a hypergeometric distribution if *p*_1 _= *p*_2 _(see the Methods section for details). We therefore compute the two one-sided-alternative *p*-values by summing up the appropriate tails of the hypergeometric distribution.

#### Measuring the difference in the quality of sites

We offer two ways to measure the difference in the quality of the sites. Our null assumption is that the scores of the sites from the two sets form two independent samples from the same, unknown, distribution. A plausible alternative is that one distribution tends to produce better scores than the other, or more precisely, that it is stochastically greater. The Mann-Whitney test is a non-parametric test that is optimized for testing the alternative that one distribution is a shifted version of the other. While we cannot assume this particular alternative here, this test should still be a reasonably good choice.

Alternatively, SADMAMA can perform a t-test of the difference between the two averaged scores. However, if the motif length *l *is not very large, the score distribution can be very far from normal (e.g. [19]). Since the t-test relies on the normal assumption, it should be taken with a grain of salt here (in a future release we hope to provide a test of the validity of the assumptions required by this t-test). Since the match scores can be repeated, especially when *l *is small, we are often forced to use the tied version of the Mann-Whitney test. This becomes important when the overall number of matches in at least one of the sets is rather small (say ≤ 10). In this range, the use of the normal approximation to the Mann-Whitney test is generally discouraged and exact calculation should be used. The latter are significantly more costly for the tied case than for the no-ties case. By default, SADMAMA decides on its own which method to use when estimating the significance of the test. If the samples are sufficiently large it uses the normal approximation. Otherwise, it uses exact methods to evaluate the significance of the test. If no-ties are present, it relies on Harding's exact algorithm [20], while if there are ties, it uses a naive dynamic programming approach written by Niranjan Nagarajan.

Keep in mind that if one tests for a difference in the quality of the sites in addition to the frequency of sites, then you should, in principle, adjust for multiple testing. Note that in general we cannot assume that these two tests are independent of one another.

#### The bootstrap approach

SADMAMA offers a bootstrap [21] inspired set of tests as an alternative to the simplified models described above. Bootstrap is a "plug-in" method: to estimate some parameter of a complex distribution we conceptually plug into the appropriate formula an approximating distribution that is typically derived from a small sample of the original distribution. It is often the case that even after plugging in the simplified, approximating distribution we still need to resort to Monte Carlo methods to estimate the target parameter. These methods work by generating random samples from the simplified distribution, computing a relevant statistic, and finally estimating the parameter of interest from all these samples of the statistic. In our case the complex distribution is the one which generated the two sets, which is not really well defined, and which, in particular, does not yield additional samples. Our model of the simplified distribution is that the two input sets are generated by sampling (with replacements) contiguous blocks or substrings of *b *letters from some joint pool. SADMAMA's default assumption is that this joint sequence pool is simply the concatenation of the two input sets. The parameters we are after, are *p*-values of the statistics that measure the differences in the quality and quantity of sites between the two sets. In particular, SADMAMA can keep track of the difference in site density as well as the difference in mean site score between the two sets.

For example, to evaluate the difference in site frequency between the two input sets, SADMAMA first finds this difference. It then creates a large number of "bootstrap images" of the two input sets by resampling *b*-long substrings, or blocks, from the concatenated original sets. Using these Monte Carlo images, SADMAMA generates an empirical distribution of the difference in site density, from which we can readily deduce an "empirical *p*-value" of the difference in site density between the original input sets. With increasing number of bootstrap images, this empirical *p*-value should better approximate the *p*-value defined by our simplified distribution. The latter should in turn be a reasonable approximation of the "real" *p*-value defined in terms of the original, complex distribution.

While in principle this is how SADMAMA implements bootstrapping, there is one more issue we had to address. Generally, when resampling the input sets we would like to avoid using blocks that are too long, as those hinder "proper mixing". The problem with smaller blocks is that, especially when the block size *b *is smaller than the motif width, essentially all the original sites that were present in the joint pool are obliterated during resampling. This is not an issue if both sets consist only of the "background signal". However, if the two sets are highly enriched with sites, yet in the same way, this kind of bootstrap test might erroneously report significant difference in site density. The reason is that the difference between the two enriched sets might be significant when compared with the typical difference between sets that were essentially made to look like background sets by inadvertently destroying all the sites (see the section on Applications of SADMAMA below).

To avoid such false positives, we implemented in SADMAMA a novel "site-protected bootstrap" approach. It is designed to allow us to sample from the original sites even if the block size is smaller than the motif width. More explicitly, each randomly chosen block might be extended so as to avoid chopping sites. The decision whether or not to extend, or protect, each such block is made in a probabilistic and independent fashion. The length of the extension is the minimal one necessary to avoid chopping any site that started (or, ended if reverse complement search is considered) within the *original *block. The probability of extending a block is defined so as to make the expected frequency of sites in the combined bootstrap sets the same as the frequency of sites in the original pool. See the Methods section for details on the technique and the section on Applications of SADMAMA for examples of its utility.

In general we found that the bootstrap tests follow closely the simplified models based tests. While the bootstrap approach might seem more attractive as it is not derived from an arguably overly simplified model, it takes considerably longer to run to get reliable estimates.

### Applications of SADMAMA

#### Stable vs. unstable ARSs in *mcm1-1 *mutant

Mcm1 is a transcription factor that has been shown to affect the efficiency of replication origins both directly, by binding to replication origins ([22,23]), and indirectly, by regulating the expression of several factors of the pre-replication complex [24]. The *mcm1-1 *point mutant has been shown to exhibit DNA replication defects in *S. cerevisiae *[25].

Functionally, ARSs are divided into two types based on their ability to function in *mcm *mutant strains such as *mcm1-1*. Stable, or A-type ARSs function efficiently in both wild-type and mutant cells, whereas unstable, or B-type ARSs function poorly in *mcm *mutant backgrounds.

Several previous studies have shown a relationship between replication initiation and local transcription patterns ([26,1]). More precisely, in [1] we show that transcriptional interference correlates with reduced ARS activity in that 80% of ARSs located in such transcriptionally active zones are B-type, whereas only 45% are B-type in transcriptionally inactive zones (see Table 1). While transcriptional interference is statistically significant, it is clearly not the sole determinant of ARS activity under this unfavorable condition (*mcm1-1 *mutant).

**Table 1:** Classification of A-type and B-type ARSs based on local transcription patterns

Transcription pattern	ARS efficiency in *mcm1-1*	
	Unstable (B-type)	Stable (A-type)

→ • → ← • ← → • ← (+)	32	8
← • → (-)	13	16

Arrow represents direction of transcription. Filled circle represents location of ARS. (+) = transcriptional interference; (-) = no transcriptional interference. Using Fisher's exact with a test two-sided alternative, independence is rejected at 0.0018.

Higher affinity for Mcm1 has been suggested to be a distinguishing feature for telomeric ARSs that are constitutively active in the *mcm1-1 *mutant [23]. In particular, footprinting assays identified a set of binding sites of Mcm1 in these ARSs. Interestingly, many of these sites can be considered as "half sites", in that they match only half of the canonical Mcm1 binding site. It is thus tempting to conjecture that stable or A-type ARSs would, in general, exhibit better (possibly half) binding sites for Mcm1 than B-type ARSs. Similarly, it was suggested that Abf1 may also have a positive effect on the formation of the pre-replication complex (e.g., [27-29]) and is therefore another natural candidate for our differential binding affinity analysis.

To test these hypotheses we applied SADMAMA to analyze the difference in the quality and/or number of these PWM matches (MCM1, half-MCM1, ABF1) between the stable and unstable sets of transcriptionally active, or (+), ARSs. SADMAMA did not find statistically significant variation in the quality or the number of MCM1 matches (see the Methods section for more details). However, it found that the stable set has more half-MCM1 sites (threshold 0.05% *p*-value 0.007) or alternatively better sites (threshold 0.1% *p*-value 0.002). Similarly the stable set has more ABF1 sites (threshold 0.1% *p*-value 0.002), or alternatively better sites (threshold 0.5% *p*-value 0.003). These *p*-values should be adjusted for the fact that we considered 3 thresholds (0.5%, 0.1%, and 0.05%) but they are still significant at the 5% level even after this adjustment. We had no reason to suspect a difference in ACS matches, and indeed SADMAMA's corresponding *p*-values were unimpressive even before the multiple testing adjustment. The *p*-values reported above were generated using the hypergeometric or Mann-Whitney tests. However, Monte Carlo site-protected bootstrap tests gave very similar results (block size *b *= 12).

For comparison we also applied SADMAMA to study the difference in these PWM matches between the stable and unstable set of transcriptionally inactive, or (-), ARSs. This time no significant *p*-values were reported. Taken together these results support the hypothesis that half binding sites of MCM1 as well as sites of ABF1 in flanking regions of an ARS may protect the ARS from incoming transcription traffic ([23,29]) but would have little influence on the stable ARSs that are not subjected to transcriptional interference.

#### *S. kluyveri *vs. *S. cerevisiae *ACS

To get a better understanding of DNA replication initiation in *S. cerevisiae*, we performed a screen to isolate fragments of *S. kluyveri *DNA that have ARS function in *S. cerevisiae*. Specifically, we cloned random fragments of *S. kluyveri *DNA into an ARS-less vector, transformed the resulting genomic libraries into *S. cerevisiae*, and isolated 46 distinct plasmids which showed ARS activity (*S. kluyveri *ARSs below). Using the same protocol we also isolated 36 native *S. cerevisiae *ARSs (*S. cerevisiae *ARSs below). Naturally, one wonders what confers *S. cerevisiae *replication activity to these *S. kluyveri *DNA segments. In particular, we should compare them to our native *S. cerevisiae *ARSs, and SADMAMA is a convenient tool for that.

We looked for significant differences between the *S. kluyveri *and the *S. cerevisiae *set of ARSs in terms of binding sites of several auxiliary DNA binding factors that are known to be associated with replication initiation: Mcm1, half sites of Mcm1, Rap1, and Abf1. SADMAMA did not find significant variation in any of these. However, surprisingly SADMAMA did find significantly more ACS matches in the *S. kluyveri *ARSs than in the *S. cerevisiae *ARSs (threshold 0.05% *p*-value 0.0004). Interestingly, when it came to quality of sites, SADMAMA reported that the *S. cerevisiae *ARSs had better sites (threshold 0.05% *p*-value 0.008). This analysis suggests that the *cerevisiae *replication initiation machinery can function with multiple weaker ACS sites such as the ones we found in the *S. kluyveri *ARSs as well as with the fewer but better native sites. This conjecture is consistent with a recent related analysis of native *S. cerevisiae *ARSs that contain multiple ACS matches [18].

It is interesting to note that the *S. cerevisiae *ARSs (the overwhelming majority of which lie within intergenic regions) have the *same *AT content as the general *S. cerevisiae *intergenic average: 66%. However, 69% of the *S. kluyveri *ARSs are made of AT, which is significantly *higher *than the 58% AT content for general *S. kluyveri *intergenic regions. Since the ACS matrix is itself AT-rich, we asked whether these *S. kluyveri *ARSs owe their functionality only to a local spike in the AT content. Using SADMAMA we addressed this question in two different ways. First we compared the *S. kluyveri *set of ARSs with 10,000 random permutations of itself. In all of those 10,000 comparisons SADMAMA found that the permuted set had a statistically significant smaller number of sites (see the Methods section details).

Similarly, we used SADMAMA to compare the ACS PWM with 4,000 column-wise random permutations of itself. In only 19 of these 4,000 comparisons did the *S. kluyveri *set have more sites of the permuted PWM than the original PWM (keep in mind that the ACS PWM is very AT rich itself so many of the permutations should not look that different from the original PWM). Taken together, these two tests indicate that there is more "ACS information" in our *S. kluyveri *ARSs than their AT content alone yields.

#### Site-protected bootstrap

To test the utility of the "site-protected" bootstrap option in a realistic setting we generated two sets of *S. cerevisiae *ARSs by arbitrarily splitting a subset of the confirmed ARSs in the DNA replication origin database OriDB [30], into two roughly equal sets: an "even" and an "odd" one. Given the arbitrary nature of the split between the sets we expect that there should not be a substantial difference in ACS sites between the two. Note, however, that both sets are highly enriched with ACS sites (see the subsection on Bootstrap tests in the Methods section for details).

Using the hypergeometric test SADMAMA found, as expected, no significant difference in ACS site-frequency between these two sets. However, when using the naive bootstrap approach with block size *b *≤ 15 SADMAMA consistently reported that one of the sets is significantly enriched in sites. On the other hand, when the "site-protected" bootstrap option was turned on, SADMAMA consistently found the difference in site-frequency between the two sets to be insignificant (even for *b *= 1).

**Figure 1:**
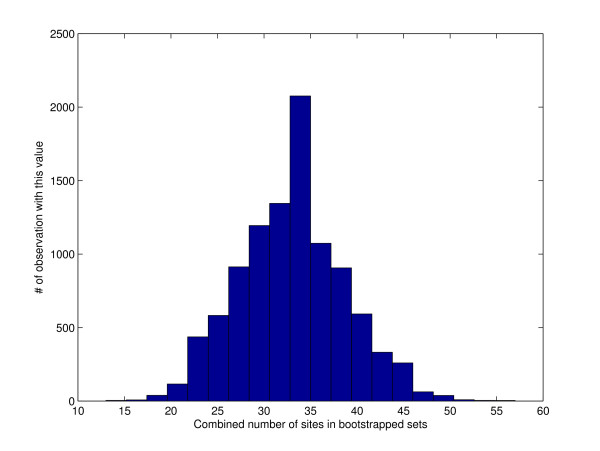
**A histogram of the total number of sites in 10,000 naively resampled pair of sets**. The mean total number of sites is 33. For comparison, there are 173 sites in the input pair of sets. Here *b *= 10 (see the subsection on Bootstrap tests in the Methods section for additional settings).

**Figure 2:**
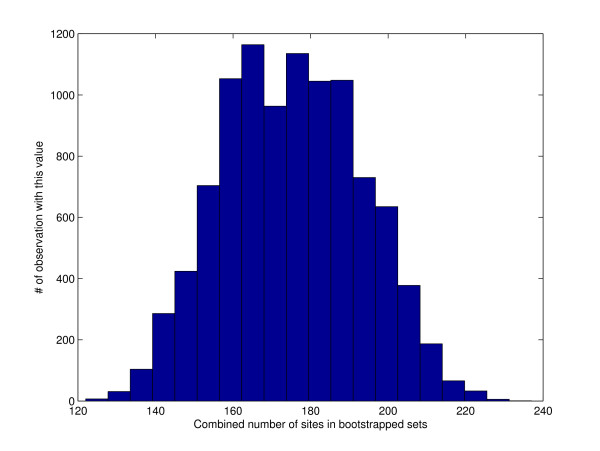
**A histogram of the total number of sites in 10,000 site-protected resampled pair of sets**. The mean total number of sites is 175. For comparison, there are 173 sites in the input pair of sets (*b *= 10).

A look at Figure 1 and Figure 2 explains what is going on. The total number of sites in a naively resampled pair of sets is typically significantly smaller than the number of sites in the input sets whereas the site-protected option manages to be consistent with the total number of sites in the two input sets. Note how the range of values observed in Figure 1 is significantly smaller than the range observed in Figure 2. This smaller range suggests that normal random fluctuations observed when sampling from the latter distribution might be considered very significant when compared against fluctuations observed when sampling the first distribution.

#### Exploratory data analysis with SADMAMA

SADMAMA can also be used to study potential enrichment of binding sites within a single set. For example, we studied whether the set of all 325 confirmed *S. cerevisiae *ARSs taken from OriDB [30] shows enrichment of PWM matches for any one of the 79 *S. cerevisiae *transcription factor PWMs defined by Morozov and Siggia [31]. For each such PWM SADMAMA tested whether the frequency of sites in the ARS set is significantly higher than in ARS-less *S. cerevisiae *intergenic file (see the Methods section). After adjusting for multiple testing, the only PWM that showed such statistically significant site frequency enrichment is the one representing *FKH2*. Interestingly, Fkh2 is known to interact with Mcm1 to form a complex that regulates the cell cycle dependent expression of the CLB2 cluster in G2/M phases in S. cerevisiae [32].

Upon closer inspection of the set of ARSs we found that many of the sites SADMAMA attributed to *FKH2 *overlapped with ACS matches and indeed aligned properly the matrices are quite similar. Moreover, after masking the high scoring ACS matches in the set of ARSs SADMAMA found the *FKH2 *site enrichment insignificant (see the Methods section for more details). Finally, the actual binding location data for *FKH2 *[33] exhibits no significant correlation with ACS sites located in confirmed ARSs.

This result seems somewhat disappointing given that the enrichment of *FKH2 *sites can apparently be explained by the obvious enrichment of ACS sites. However, SADMAMA's results still leave us with a potentially interesting question: does the similarity between binding sites of *FKH2 *and the ACS have any biological importance? Analysis of the literature suggests a positive answer is conceivable. Specifically, when overexpressed, Fkh2p is known to have a negative role in silencing the silent mating-type cassette *HMRa *in *S. cerevisiae *[2]. Moreover, it is known that ORC binding to the ACS is associated with the chromatin silencing process at this locus (e.g. [34-36]). Consistent with the similarity we observed in their binding sites, it is tempting to conjecture that *FKH2 *might interfere with the chromatin silencing process by offering some form of competitive binding to ORC. Since the interference of Fkh2p was observed when it was overexpressed, the lack of support from the location data [33] does not rule out this conjecture.

## Conclusion

SADMAMA offers a computational solution to a novel problem: does one set of sequences have a statistically significant increase in the number and/or the quality of sites of a given PWM than another set. Note that setting the second set as a large background set SADMAMA can also be used to assign significance to matches in a single input set. SADMAMA implements two types of tests: one type is based on simplified sequence models while the other relies on bootstrapping and as such might be preferable to users who are averse to simplifying models. Generally, when resampling the input sets we would like to avoid using blocks that are too long, as those hinder "proper mixing". However, as we show, a naive resampling procedure using shorter blocks can bias the tests. SADMAMA implements a new stochastic feature, which we term site-protected resampling, and which successfully solves this problem.

SADMAMA's utility is demonstrated here by offering a plausible explanation to the differential ARS activity observed in our previous *mcm1-1 *mutant experiments [1], by suggesting the relevance of multiple weak ACS matches to efficient replication origin function in *S. cerevisiae*, and by suggesting an explanation to the observed negative effect *FKH2 *has on chromatin silencing [2].

To the best of our knowledge, we are the first to present a tool for studying the difference in matrix matches between two sets. Very recently, and independently of us, Robin et al. posed the analogous problem in the context of pattern representation of a motif [11]. Our hypergeometric test derived from our binomial modelling of the number of sites is somewhat similar to their binomial test, which is derived from a Poisson model. However, since we deal with matrices, we also study the difference in quality of sites which they do not. SADMAMA also offers a bootstrap approach which is not discussed by Robin et al. Finally, we provide a computational tool while they describe statistical tests.

We identified several ways to improve and expand SADMAMA's current set of features. To name a couple, SADMAMA currently assumes that the input sequences are independent which therefore excludes it from analyzing phylogenetically related sequences. Given the increased availability of related genomes, extending SADMAMA to handle such cases is highly pertinent. Similarly, for some cases one can argue that a more appropriate motif sites model is that each sequence is endowed with a small, say Poisson drawn, number of sites. Currently, SADMAMA fails to correctly handle this model if there are significant differences in the length of the sequences, and extending it to address this model as well is highly desirable. Finally, helping the users with analyzing multiple tests when such are specified could increase SADMAMA's utility. For example, when more than one site-threshold is considered, or when both the frequency and the quality of sites are examined.

## Methods

### Hypergeometric test

The abstraction of our binomial model for the number of sites in each of the input sets coupled with our null assumption that *p*_1 _= *p*_2 _= *p *is as follows. Suppose *X *is a binomial *B*(*n*, *p*) random variable and *Y*, which is independent of *X*, is *B*(*m*, *p*) (same *p*). Conditioned on *X *+ *Y *= *k *(total number of sites in both sets), *X *has a hypergeometric distribution *H*(*n*, *m*, *k*):

$$\begin{array}{lll} P(X=l|X+Y=k) & = & \frac{P(X=l,Y=k-l)}{P(X+Y=k)}\\ & = & \frac{\left(\begin{array}{c} n\\ l \end{array}\right)p^{l}{\left(1-p\right)}^{n-1}\left(\begin{array}{c} m\\ k-l \end{array}\right)p^{k-l}{\left(1-p\right)}^{m-(k-l)}}{\left(\begin{array}{c} m+n\\ k \end{array}\right)p^{k}{\left(1-p\right)}^{n+m-k}}\\ & = & \frac{\left(\begin{array}{c} n\\ l \end{array}\right)\left(\begin{array}{c} m\\ k-l \end{array}\right)}{\left(\begin{array}{c} m+n\\ k \end{array}\right)}. \end{array}$$

Thus the *p*-value of an observed value *X *= *x *against the one sided alternative *p*_1 _> *p*_2 _is

$$\sum_{l=x}^{k}\frac{\left(\begin{array}{c} n_{1}\\ l \end{array}\right)\left(\begin{array}{c} n_{2}\\ k-l \end{array}\right)}{\left(\begin{array}{c} n_{1}+n_{2}\\ k \end{array}\right)},$$

where *k *is the combined number of sites observed in both sets, and *n*_1 _and *n*_2 _are the number of feasible site locations in the input sets (slightly less than their lengths due to "edge effects": a site cannot begin too close to a sequence end). Technically we use Catherine Loader's carefully implemented package [37] to execute the crux of the computation.

### Site-protected bootstrap

The success of the site-protected bootstrap option in SADMAMA hinges on its ability to set a "reasonable" value for *α*, the probability that SADMAMA protects the block (more precisely, it minimally extends the randomly chosen block so as to include all sites that started within that original block). SADMAMA's strategy is to choose *α *so that the expected total frequency of sites across the two sets is close to (ideally the same as) the site frequency *ν *in the sample pool. By default, the latter is the concatenation of the two input sets. Setting *α *= 0 amounts to the naive resampling approach as no block will be extended. As we saw in the section on Applications of SADMAMA this tends to generate samples with site frequency <*ν *(Figure 1). These site-poor samples can in turn inflate the overall significance of the test. On the other hand, setting *α *= 1 amounts to protecting every sampled block. This setting does not take into account potential new sites appearing across the seams of sampled blocks and therefore it tends to generate samples with site frequency > *ν*. This in turn could yield a test which is too conservative.

SADMAMA sets *α *before the main resampling loop begins. Our goal is to set *α *so that the frequency of sites in an infinitely long sequence constructed from the site-protected resampling procedure will be exactly *ν*. In reality we settle for a fairly long sequence generated by this procedure. But how long is long enough? Clearly, this length should be a function of *ν*: the smaller *ν *is, the longer the training sequence needs be. More generally, how can we be confident we have a "reasonable" estimate of a Bernoulli success probability *ν*? One way is to generate sufficiently many trials so that the size of our confidence interval for *ν *is a small fraction, *γ*, of *ν *(*γ *= 0.05 is SADMAMA's default). Here we aim at estimating a site frequency which is roughly *ν *so using a Wald (normal) confidence interval implies

$$c\sqrt{\nu (1-\nu )/n}\leq \gamma \nu ,$$

where *c *is a small factor determining the size of the confidence interval (*c *= 3 by default), and *n *is the resampled sequence length we seek. It follows that we should set

$$n\geq {\left(\frac{c}{\gamma }\right)}^{2}\frac{1-\nu }{\nu }.$$

In practice, to keep runtime and memory requirements under control SADMAMA caps the size of this sequence using a compilation time parameter (currently set at 10^6^).

One approach to setting *α *would be to design a binary search keeping in mind the stochastic nature of the resampling procedure. The main downside of such an approach is that generating and then scanning a large sequence for sites can be time consuming. While one can imagine various tricks to speed up this process we chose a different shortcut.

As mentioned above, there are two types of sites in our site-protected resampled sequence of length *n*. Type I sites are sites that are entirely contained in a resampled block, i.e., they also appear in the original sample pool. Type II sites, are newly generated sites that span two or more resampled blocks. These resampled blocks are adjacent in the resampled sequence but not in the original sample pool. Let *K *be the (random) number of blocks required to generate the resampled sequence and let *B*_*i *_denote the *i*th random block. Then the random number of sites, *S*_*α*_, is given by

$$S_{\alpha }=\sum_{i=1}^{K}1_{\left\{B_{i}\text{ has a type I site}\right\}}+1_{\left\{\text{a type II site starts in }B_{i}\right\}}.$$

To simplify our derivation, we now assume that the block size *b *is less than the motif length *l *and we ignore the fact that the last block is typically truncated. In this case, the event {*B*_*i *_has a type I site} only occurs if originally a site starts in block *B*_*i*_, with probability ≈ *b ν*, and the block is protected, with probability *α*. It follows that

$$E(1_{\left\{B_{i}\text{ has a type I site}\right\}})\approx b\nu \alpha .$$

The number of blocks we extend is negatively correlated with *K*. However, to first order, $E(K)=\frac{n}{b}+O(l\nu \alpha )$ and, as *lνα *is typically negligible compare to $\frac{n}{b}$, we can assume $K\approx \frac{n}{b}$ is roughly constant. Therefore,

(1) $$E(S_{\alpha })\approx n\nu \alpha +\frac{n}{b}\mu$$

where $\mu =E(1_{\left\{\text{a type II site starts in }B_{i}\right\}}$ is the probability of a new, shared-block, site.

**Figure 3:**
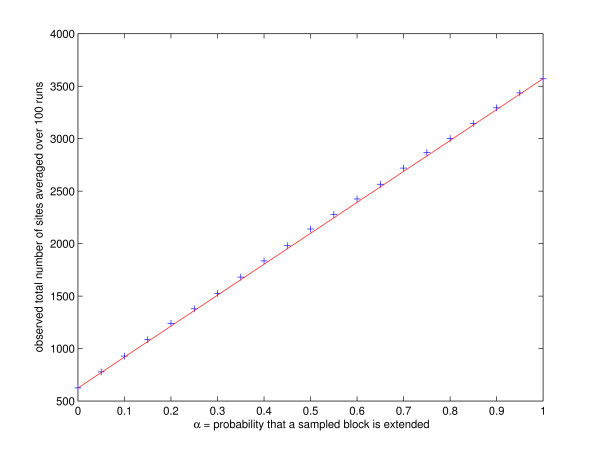
**The expected number of sites as a linear function of *α***. Average total number of sites in the sequence per *α*, the probability that a sampled block is extended. Site threshold, background file and all similar settings were as described in the subsection on Bootstrap tests in the Methods section. The average was taken over 100 random resampled sequences of length *n *per each value of *α*.

The exact form of (1) is not that important to us here, as is the fact that the right hand side is a linear function of *α *(see also Figure 3 for an empirical demonstration). We therefore estimate *E *(*S*_*α*_) for *α *= 0 and *α *= 1 by generating corresponding resampled sequences of length *n *and counting the number of observed sites *m*_0 _and *m*_1 _respectively (these resampled sequences are solely generated for the purpose of determining *α *and are not further used in SADMAMA's main bootstrap tests). SADMAMA then relies on linear interpolation to set

$$\alpha =\frac{n\nu -m_{0}}{m_{1}-m_{0}}$$

so that *E *(*S*_*α*_) = *nν*. Note that if $\nu <\frac{m_{0}}{n}$ SADMAMA sets *α *= 0 and throws up a warning that random shuffling of blocks creates more sites than there were to begin with. Similarly, if $\nu >\frac{m_{1}}{n}$ SADMAMA sets *α *= 1 as that is the highest density of sites you can get with this recipe.

### Background model

In all the tests we report, we used SADMAMA's default setting of a 3rd order Markov background model. Unless otherwise stated, the training file from which this model was learned was our "standard *S. cerevisiae *intergenic file". This file was generated by removing from the *S. cerevisiae *genome downloaded from SGD [38] all protein and RNA coding sequences including tRNA, rRNA, snoRNA, snRNA and other presumably irrelevant elements such as LTR, and repetitive sequences. We also generated an "ARS-less *S. cerevisiae *intergenic file" by removing all 325 OriDB-confirmed ARSs [30] from our standard *S. cerevisiae *intergenic file.

### Stable vs. unstable ARSs in *mcm1-1 *mutant

The ARSs we identified in our *mcm1-1 *screen were much longer than the typical size of confirmed ARSs in OriDB [30]. To perform our statistical analysis we therefore restricted our attention to what we conjectured to be the core of each of these ARSs. Specifically, we picked the best ACS match in each of these ARSs, as predicted by [39], and considered only the 200 bases on each side of this match. Similar lengths were explored giving essentially the same picture. We note that two of the ARSs, one stable and one unstable, had no predicted ACS matches so we left those out for this analysis.

The ABF1 and MCM1 matrices were taken from TRANSFAC [40] via TESS [5]. Given the palindromic nature of the MCM1 sites, the half MCM1 matrix was defined by adding the reverse complement of the second halves to the first halves of the sites. The ORC matrix was taken from [39]. All matrices were adjusted using a total pseudocount of 10% added uniformly to all bases. Site thresholds were set so that 0.5%, 0.1%, and 0.05% of the words in the standard *S. cerevisiae *background file exceeded these numbers. The maximal overlap allowed between sites in this as well as all our other tests in this paper was the default 20%.

### *S. kluyveri *vs. *S. cerevisiae *ACS

Our *S. kluyveri *set of ARSs included 46 *S. kluyveri *DNA segments (defined using DpnII which is a 4-cutter restriction enzyme recognizing the sequence GATC) that conferred S. cerevisiae-ARS activity to the plasmid. The *S. kluyveri *set included 37,176 bases, 69% of which were AT. Our *S. cerevisiae *set, generated using the same protocol, included 36 sequences of *S. cerevisiae *DNA containing 29,561 bases, 66% of which were AT. The ACS matrix was again taken from [39]. Given the amount of data from which this PWM was generated, we used a reduced pseudocount of 1% added uniformly to all bases. Our ARS screen was carried out in *S. cerevisiae *and as noted the AT content of the kluyveri set was much more in line with *S. cerevisiae *intergenic DNA than *S. kluyveri *one. We therefore used the aforementioned standard *S. cerevisiae *intergenic background file. The site threshold was set to 0.05%, that is, roughly 5 in 10,000 words in the background file are above the chosen threshold (similar results were observed with the 0.1% threshold).

In our first of two types of permutation tests we ran SADMAMA 10,000 times with the *S. kluyveri *set serving as the first as well as the second input set. SADMAMA was instructed to randomly permute the second input set, which it does by separately permuting each sequence in the set. In each of these 10,000 runs the unpermuted *S. kluyveri *set was deemed to have more sites with a *p*-value ≤ 0.05.

In the second of our permutation tests we ran SADMAMA 4,000 times comparing the *S. kluyveri *set against a dummy set while asking SADMAMA to permute the given ACS matrix. SADMAMA then found the threshold so that the background file will have a rate of 0.05% sites of the permuted matrix which is the same as the percentage of sites for the original, unpermuted, ACS matrix. For each permuted matrix we keep tally of how many sites SADMAMA identifies in the *S. kluyveri *set (in this mode no tests were actually done: SADMAMA simply counts the number of sites above the threshold which it computed as described above), and we compare those counts with the number of (unpermuted) ACS sites in the same set. It is important to note that, generally, setting the threshold can be rather arbitrary. However, this is not the case when you want to compare site counts of different matrices. Therefore, to make sure the threshold is set to control "background" rather than "real" sites we used the ARS-less S. cerevisiae background file in this test.

### Bootstrap tests

From the list of 325 confirmed *S. cerevisiae *ARSs on OriDB [30] we selected all ARSs shorter than 400 base pairs. Ordering these selected ARSs according to their location in the genome, we then assigned all even numbered ARSs to the "even" set (116 sequences, 28327 bases) and all the odd ones to the "odd" set (116 sequences, 28932 bases). Using the hypergeometric test SADMAMA *p*-valued these sets' enrichment in ACS sites relative to the ARS-less *S. cerevisiae *intergenic file (see Methods section) at 3 × 10^-90 ^and 6 × 10^-74 ^respectively. In these runs SADMAMA used same ACS matrix from [39] and pseudocount of 0.1 as above. The site threshold was set to 0.01% relative to the background file which was the standard *S. cerevisiae *intergenic file.

Using the same settings, SADMAMA's hypergeometric test comparing the even and the odd sets was insignificant at 0.87 and 0.16, depending on the chosen one-sided alternative. However, using the naive bootstrap test with block length *b *= 6, SADMAMA reported that the even set is significantly enriched for ACS sites with a *p*-value of 0.0005. The difference is still significant at 0.008 for *b *= 10, and it is even significant for *b *= 15 at 0.04. With the site-protected feature turned on and *b *= 6, SADMAMA found the observed difference in ACS sites frequency to be insignificant at 0.87 and 0.13 depending on the chosen one-sided alternative. These *p*-values remained roughly the same for all other block lengths we looked at including *b *= 1. Other bootstrap settings were: site statistics are gathered set-wide, using 10,000 resampled pairs, both sets are resampled from a sequence generated by concatenating the two input sets (-tests freqScoresGTT MC -- -MCstatScope setWide -numRandomSets 10000 -set1RandTrainFile _BOTH_ -set2RandTrainFile _BOTH_ -MCmodel bootstrap -v 0.2 -m 3 -pwmPC 0.01 -siteThresholdLearnedFrom 0.0001 nullTrainFile).

### Exploratory data analysis with SADMAMA

We downloaded the set of "Phylogibbs PWM predictions" of Morozov and Siggia [31], which contains 79 predicted *S. cerevisiae *matrices. For each of these matrices SADMAMA looked for enrichment in site frequency in the set of 325 confirmed ARSs relative to the ARS-less *S. cerevisiae *intergenic file. The threshold was set to 0.05% relative to the standard *S. cerevisiae *intergenic background file, and a total pseudocount of 10% was added uniformly to all bases. The *p*-value of the FKH2 matrix is 4.7 × 10^-5 ^and the *p*-values for all other 78 matrices are > 10^-3^, which is insignificant when corrected for the multiple testing.

## Authors' contributions

UK designed the statistical tests, drafted the manuscript, implemented the software and executed the tests on the biological data. HG and JG helped with implementing the software and executing the tests, AB helped with executing the tests and drafting the manuscript, IL and JD generated the biological data and helped drafting the manuscript, BKT helped conceive the experiments and drafting the manuscript. All authors read and approved the final manuscript.
